# Comparative Psychology*Read before the Association for the Study of Comparative Psychology in connection with the Montreal Veterinary College.

**Published:** 1888-01

**Authors:** T. Wesley Mills

**Affiliations:** Professor of Physiology, McGill University, Montreal


### ART. V.—COMPARATIVE PSYCHOLOGY.*

BY T. WESLEY MILLS, M. A., M. D.

*Professor of Physiology, McGill University, Montreal.*

In entering upon the third year of our existence as a Society it has seemed to me that it might be encouraging to the older members and instructive to those who are meeting with us for the first time, to review the work of the Society for the past two years; to point out what we have tried to accomplish and what has been actually achieved.

Believing that men who had chosen comparative medicine as a career must have some real liking for those animals, at least, which are classed as domestic, if not for all creatures that breathe the breath of life, and feeling assured that a knowledge of the mental constitution of animals must prove invaluable to the veterinary surgeon in the diagnosis and treatment of the diseases of his speechless patients, in the latter part of the year 1885 I called together such of the students of the Montreal Veterinary College as were attending my own classes in physiology and suggested the desirability of forming some sort of association for the attainment of these objects. Those addressed responded to my proposal as only young men can. Soon almost every student in the college joined us. The Principal and Professors aided both, by smoothing the way and by active and cordial co-operation. A spacious and comfortable room was kindly placed at our disposal in the Veterinary College building in which to hold our meetings. As the project was tentative we did not think it well to fetter ourselves with many rules or regulations. However, on commencing our second year, we felt warranted in giving our Association a name, providing for it a constitution and by-laws, and taking such other steps as tended to show that

*Read before the Association for the Study of Comparative Psychology in connection with the Montreal Veterinary College.

organization was warranted as a natural result of growth and development.*

In order to present the history of our Association within a small space, you will bear in mind that the accounts of papers read and the discussions ensuing must appear in very condensed form ; and that the comments I have now to make on them must be few, and rather indicative of the line of investigation we have followed and should continue to pursue than as statements of established results. Naturally most of our studies, though by no means all, have been of the domestic animals, and, as was to be expected, the dog is the creature whose mental nature has been most frequently the subject of our inquiries ; and this will likely be the case in the future, also, for many reasons ; for if we can establish some conclusions regarding the psychic operations and development of any one of the lower animals, we then have more certain ground for comparison, even if we never succeed in showing that we have any warrant for interpreting the mental operations of inferior animals in terms of those of man. If we could establish a relative scale of intelligence for animals below man, much would have been accomplished. The first communication laid before the Society grew out of a paper read before the Veterinary Association by Principal McEachran. In this communication the behavior of a dog that was manifestly possessed of unusual intelligence was described in detail. Among other evidences of this were his journeys to a baker's shop to purchase food for himself. Several such cases are on record, and as I shall have occasion to bring this matter before you again shortly, it will not be dwelt upon now. In all such cases we cannot be too cautious in the explanations we adopt. Mr. Dawes, at the same meeting, sketched the history of a Cocker spaniel that, in consequence of

* Thus far the Principal of the Veterinary College, D. McEachran, has been the Honorary President, T. Wesley Mills, Professor of Physiology, McGill University, President, and W. J. Torrance, Recording Secretary. The other offices have been filled by different members of the Association, including the professors of the college.

early training, would, on request, fetch any one of six different articles.

This case led to the important inquiry: In how far or in what sense do animals understand words? In the course of the discussion following it was pointed out that dogs would answer to their names, when uttered by strangers, in opposition to the view that the animal was guided chiefly, if not solely, by the general demeanor of the person calling the dog, the tone of voice, etc. The fact that each individual of a pack of hounds will respond to his own name was also significant. The observation that, as noticed by one member, his dog would answer sometimes to names very similar, as "Dick" and "Vick," was not without parallel in the case of men and was explicable either by imperfect hearing or by inattention.

The case as instanced of a terrier that seemed to hunt equally well for rats whether "cows" or "rats" was the inciting term, did not furnish a wholly valid objection it was thought, for in all such instances the accompaniments of the utterance of the mere word were of more significance than the word itself. I shall have evidence to present to you during this year which I think will make it clear that at least many dogs really do know their names in the same sense as very young children, if not even in a higher sense.

Frequently, during the past two years, the influence of breed, of the individuality of the owner or trainer of the animal, of food and general treatment has been under discussion.

These questions are not only of the highest theoretical interest, but of the greatest practical importance. At one of our meetings certain members advanced a view favorable to a course of severity in dealing with certain horses, such, for example, as the "bucking" ponies of the prairies. The President believed that it was of the utmost importance that such a view should not be entertained by veterinary surgeons, and that efforts should be made to eradicate it from the public mind in so far as it really exists.

Most of the difficulty experienced in managing animals arises from their not understanding what is required of them, or from mental associations which have been established by previous unwise or cruel treatment. I cannot here refrain from stating the opinion of an eminent horse-trainer with whom I lately conversed. He holds that every horse should be broken and trained by some one more or less of an expert; that we expect too great a variety of performance from the same animal. Each is naturally, to a large extent, best adapted for some one kind of work—in a word, each is, to a large degree, fitted to be a specialist. But in this case a good many drivers would require to be "broken" also. The brutes are constantly suffering from the stupidity as well as the moral obliquity of man, their controller, but not always and in all respects their superior. These remarks do not apply alone to the horse or the dog. All animals must first learn that they are to be subject to man when required; but as I have always maintained the highest results are to be secured only by kindness and discretion combined with firmness. A little reflection will show why this must be so. One does not facilitate the working of a steam engine by any sort of forcible interference with the parts of the apparatus, but by supplying good fuel and duly oiling the machine where friction is greatest. So it is for man to study the mental machinery, so to speak, and provide those conditions most favorable to harmonious working; in a word, man must adapt to nature and not attempt to make nature adapt to his views. The latter he cannot do—her plan was laid before he appeared on the scene. If an animal is so stupid or so obstinate as not to yield to such treatment, then it should be abandoned; for it will not be worth any man's while to injure his own moral nature by what is really cruel treatment for the sake of the value of such an animal.

At another of our meetings Mr. Miller referred to the case of a dog that was very anxious to accompany his master, resorting to the artifice of placing himself some two miles in advance on the road usually selected. There are many such instances, and it seems impossible to explain them except by the exercise of reasoning power or some mental process closely analogous. But it must appear superfluous to contend any longer for the possession of such a faculty in the higher groups of animals at least. One of our members, Mr. Metcalf, himself the owner of a large number of dogs, referred to the fact that one of them had a great dislike of beggars, tramps and such like persons. From what I have read of similar and even more marked conduct, from much that I have seen, and especially in a young dog I now possess, I am almost persuaded that in certain dogs such hostility is inborn and, in certain cases, hereditary. Mr. Metcalf thought that the detention, without injury, of would-be thieves, as in a case he reported, was peculiar to the mastiff.

In February, 1886, Mr. John Miller read a paper on the dog, with special reference to the Scotch collie, which brought out some interesting remarks from a member who had witnessed the training and performances of these animals in Scotland. Everything went to show that the Collie dog is a specialist of marked aptitudes, the result of ages of training and selected breeding, though his general intelligence is also high.

At the following meeting Mr. Ferron reported on the intelligence of a certain bitch he had observed. The animal imitated a cat in carrying kittens and in several other particulars ; she was also remarkable in retentiveness of memory and in other respects. This case was all the more valuable a study, inasmuch as the animal had received no training whatever.

The President instanced the case of a brindle bull dog that had on several occasions found his way home, a distance of twenty-four miles, and in so brief a time as to indicate that he must have taken short cuts. Such cases suggest one of the most interesting and puzzling inquiries in the whole realm of comparative psychology : the nature of the mental processes by which animals make their way back by a diferent route to places from which they have been taken. I have given the subject considerable attention, and I hope before very long to be able to throw some new light on this vexed question. At this meeting a paper published by Dr. Packard in the *American Naturalist*, for September, 1885, on the "Origin of the American Varieties of the Dog," was read, on account of the great interest of the subject. When we consider how widely the dog has departed from all his supposed ancestors in his psychical traits we are amazed at the extent to which lower minds can be modified, we might almost say radically changed, by contact with the dominant mind of man. This being the last meeting of the session the President proposed certain subjects for study during the summer. These were put in the following form:

To what extent have the lower animals imagination? What animals dream? The persistence and modification of instinct. Is there a "homing instinct" or a "sense of direction" peculiar to animals? What groups of animals understand mechanical contrivances and which can use tools? How far do the minds of animals become modified by contact with man? Have any animals a special aptitude or a peculiar faculty for determining where water is to be found? The special senses of the lower animals compared with those of man; feigning, catalepsy, etc, in the lower animals; a moral sense in animals below man.

It is not to be understood that our attention was devoted exclusively to the dog during our first year of existence as a Society; but it has appeared to me best to give a sketch of our investigation of each animal separately; so I now continue the account of our study of the dog during last year. Before doing so it may be mentioned that the session was opened by the election of officers and the delivery of an address by the President, in which many of the topics proposed for special study at the close of the previous year were reverted to, and the objects of the Society indicated. As this address has been published* and copies of it are already in the hands of most of the older members, I shall not further refer to it than to say that the subject has

* "Comparative Psychology: Its Objects and Problems," *Popular Science Monthly*, March, 1887.

attracted attention generally, and its treatment, as was expected, has received some criticism.*

To return to our friend the dog. Early in the session Mr. Simpson made an important communication, the result of a careful study of a blind Bohemian dog. He had proved conclusively that this animal understood his name and also many other words such as "sneeze," "bark," etc. The dog had been blind for two years, but had so made use of his remaining senses and his mental faculties generally that he seemed, except in special cases, but little worse off than before. He recollected well the location of stable objects and was able to make his way successfully through the business portion of a city of considerable size.

This paper led to much interesting inquiry, and light was thrown on the subject by comparison with blind men. Several members referred to cases of the latter whose history was known to them. The President thought that there was no doubt that the results, both in men and the lower animals, were dependent not only on greater acuteness of the other senses, but on the greater amount of attention paid by the mind to the data furnished by the former. It was to be remembered that improvement in the senses, whether in the blind or others, was largely to be referred to the brain itself. This was especially clear on studying blind persons. The progress made even in walking under difficulties was owing, in the most successful cases, in great part, to superior brain development. The subject is of wide scope and of the very highest interest, but we cannot enter upon a further discussion of it now. At our January meeting Mr. Pease reported some observations on a black-and-tan bitch. He had proved to his entire satisfaction that this animal understood the meaning of certain words perfectly well, in so far as could be judged by her actions. Thus she never confounded such words as "breakfast," "dinner," "supper." It will be seen that we have given the question of the extent to

* *Science*, vol. IX., Nos. 217, 222.

which the dog understands words, a good deal of examination. It merits the closest study, for unquestionably the magnitude of the gap between man and the lower animals is owing to the capacity of man to use, and his actual employment of, language. But I must repeat what I said in my last year's address, that it is more than likely that we much under-estimate the capacity of animals to communicate with each other by a language of their own.

Unlike the dog, the cat has received very little attention and consideration from man. There are many reasons for this neglect, but not least in significance is the fact that puss is no flatterer; the dog adapts himself to every caprice and whim of his master, but the cat is always herself. To understand her thoroughly, to see her at her best, she must be manipulated as a delicate piece of mechanism and treated in the very kindest fashion. When so dealt with the cat proves to be by no means only a comparatively untamed embodiment of certain strong instincts. I have maintained, and supported the opinion by some evidence, that the intelligence and possible good qualities of the cat have been much under-estimated.

The same may be said of another animal, that has been not only neglected by those interested in the study of animal intelligence, but misrepresented in general. I mean the pig. What would the dog be to-day, if he had, for hundreds of years, been valued only for his flesh, and kept exclusively to be fattened for food? The hog is charged with being dirty, stupid and obstinate. Why should an animal, over-burdened with flesh and fat, and consequently a sufferer from the heat of summer, be so much blamed for betaking himself to a pool even if muddy? Man is largely responsible for enforcing conditions involving filth on the hog. That this animal is not lacking in intelligence has been shown by his having been taught to hunt like a dog,* and by an interesting case, reported to our Association by Mr. Frank Miller. The animal was of the Chester White breed; had been trained at the age of four months;

* "Animal Intelligence" by G. J. Romanes, p. 339.

knew his name; would dance to music; go seek when told, lie down, and obey other commands. You will at least agree that the hog is worthy of further consideration at our hands. Circumstances were mentioned by one of our members which pointed very strongly to the possibility of hogs having hibernating capacity. This subject is of great physiological interest, and not without its bearings on comparative psychology; any new light on the subject, so far as animals, especially, are concerned, would be very welcome.

We have had a few communications on the intelligence of our domestic grazing animals. Mr. Torrance had observed that sheep had acted, on different occasions, as if they were aware of the approach of storms still distant. Their behavior, in seeking shelter, had been coincident with changes in the barometric pressure.

Numerous reports from the sites of recent earthquake shocks by observers of unquestionable reliability have shown that many different kinds of animals were sensible of something abnormal, which caused in them manifestations of uneasiness or fear, some seconds before anything unusual was noticed by man. As I hope to show on some other occasion, such indications of acute sensibility or close observation, throw much light on certain vexed questions in the science of comparative psychology.

Among wild animals we have had several short but interesting communications on the gopher of the prairies; also a very carefully prepared paper from Mr. Harris, giving the results of his own investigations of the beaver and his work in the Canadian Northwest.* Confirmations of these observations and additions thereto were offered by another member who had much experience of life on the prairies. It becomes very clear that the beaver is not only an animal of strongly pronounced instincts, but of great capacity to adapt itself to circumstances (plasticity of instinct, etc.) I again raise the question: What is the mental difference between the performances of the beaver and those of a man with marked genius for engineering operations, apart from

* This paper has since been published, by request of the editor, in the *Canadan Journal of Fabrics.*

all training? Only prejudice can prevent us seeing that this is a case of the highest suggestiveness; and it is, to me, replete with instruction. The time would fail me to attempt to even indicate to you how far-reaching is such an inquiry. I cannot help thinking that man would both understand himself better and have a truer insight into the inner life of the so-called inferior animals if he could get rid of some of his conceit, and regard himself rather as of than apart from the rest of the animal creation. The achievements of the nineteenth century are great; so also is its conceit.

The study of the apes and monkeys, on account of undoubted physical and mental resemblances to man, is na'urally of the greatest interest. Accordingly a communication from Mr. Clement on a monkey he had kept under observation, was welcomed by the Association. This creature's curiosity, observing powers, retentiveness of memory, and confidence in his owner, in contrast with a shyness towards strangers, were pronounced. His power of imitation, it was thought, had much to do with his mental progress. The superiority of this monkey, as in other cases, was evidenced by his capacity for education. As Mr. Clement well observed, there was much in the creature's behavior that suggested the child. The President had, in the case of this individual, verified Darwin's statement that monkeys have an instinctive fear of snakes. When this animal was offered a dead snake in a paper bag he cautiously peeped in and then ran away in terror; nor could he be induced to go near the bag again. I may mention incidentally, that there is now in Central Park Menagerie, New York, a remarkable chimpanzee, of an intelligent expression of countenance so human-like as to be positively startling. If now he could but stumble on speech; what then? It seems not unlikely that the superiority of the monkey's brain over that of other animals may be owing in part to the use of the forelimb a;s a hand. It has even been suggested that the greater brain-weight of man compared with that of woman may, in part, be the result of his more pronounced muscular development.

We have endeavored to throw some light upon the question as to whether any animals have a special aptitude in finding water. There is a certain amount of evidence in the affirmative as regards frogs, turtles and allied animals. A member well acquainted with life on the plains referred to the fact that thirsty travelers are accustomed to follow a "buffalo rut" in the confident hope of finding water. We need more exact information on such subjects very much.

Turning now to that most faithful servant of man, the horse, we must confess to having made less progress than in the study of the dog; and I would suggest that the reason is partly to be found in the very fact, that this animal is a servant rather than a companion of man. The whole nature of the horse is restrained and modified so that he may be adapted to human uses, and as a result we fail to see him, in his true nature, as a freely developing animal. The horse has become to a large extent a living automaton; as such, he is an interesting evidence of the dominance of one intelligence and will over another; but the real nature of the animal is, in consequence, much obscured. We have had, however, many interesting communications on the horse. At an early meeting of the Association Mr. Dawes presented the formulated opinions of an expert trainer. Among the most important of these are the following: Horses know their names; they recognize each other after long separation; the development of high speed trotting is largely dependent on the intelligence displayed in the training; horses exhibit judgment in the choice of track and in jumping hurdles; some horses neigh when the groom is seen at the feed-box or the water-tap; some even attempt to turn the tap; horses frequently endeavor to throw vexatious or unskillful riders. I shall be glad to communicate to the Association the results of an important interview I had during the past summer with an eminently successful trainer. His experience confirms and amplifies views I have on more than one occasion expresse before you.

Several members stated that they had noticed that horses have a special dread of bears, and could scent them at a great distance. Some horses were also afraid of a fur coat. No one, however, seemed to be able to explain why this fear should attach to bears more than to other ferocious animals; one would expect, in fact, that there would be much more danger to the horse from wolves than bears. Is this the remnant of a once powerful instinctive fear? At a later meeting Mr. Dawes continued the subject of equine psychology. He explained that a mare, in his possession, had learned to overcome, in succession, different mechanical contrivances, such as buttons, fastenings, etc., which had been placed on the feed-box to prevent its being opened. Mr. Dawes also instanced the behavior of one of his horses which showed considerable intelligent association of ideas. This animal was accustomed to being driven to the railroad station, and on certain occasions, on hearing the whistle of the approaching train, had started off on his own account, and after the train had left he had returned home. The ability of horses to remember incidents, sometimes trivial, after the lapse of years was testified to by several members.

Mr. Ferron has also favored us with some interesting facts in regard to the intelligence of trotting horses; and the Principal of the College has pointed out instances of equine sagacity, of a very striking kind. Horses had even come to the College hospital for treatment of their own accord. While all our domestic animals amply repay good treatment, of none is this more true than of the horse. To take advantage of this animal's gentle, sensitive, plastic nature to subject it to abuse is the part only of a savage and not of a civilized human being. It is not to be forgotten that ill treatment of the brutes reacts on the moral nature of the man that is guilty of it; in injuring them he injures himself far more.

But the time fails me. This is necessarily a very inadequate account of our work up to the present—a mere sketch—but I hope it may suffice to encourage old members and to arouse the interest and enthusiasm of those now entering to fill the places of the men who have left us, and whose efforts in this cause, we must gratefully remember. There is one thing which cannot in any way be represented to others, and that is the delight we have experienced in meeting together to discuss the inner and, unfortunately for us, so much hidden life of those beings that we have learned to regard with more and more respect and to consider fellow-creatures. It will take some time to educate the public mind up to the point of realizing how much these animals are really deserving of serious, respectful consideration. To the enlightened veterinary surgeon must we especially look for an improvement of the condition of our domestic animals; and in no way can this be accomplished more effectually than by learning their true nature and making that known. We wish to reach only the truth. No cause is in the end advanced by over-statement of the facts.

Sensible rather of how much is still to be done, than satisfied with our past progress we renew our inquiries in the firm belief that an honest, humble search after truth will never be in vain.*

### ART. VI.—LYSSA AND THE PASTEUR FIASCO.†

BY N. E. BRILL, A. M., M. D.
*New York.*

Even in the developmental history of disease we see, curiously enough, the reflection of the struggle for existence in the human species. The most important factors in the human mental organization, which tend to keep alive those best fitted to cope with the obstacles which meet them in their struggle for life, are the instincts and their

* On behalf of the Association the President would be glad to receive, from any quarter, *accurate* accounts of such facts as are calculated to throw light on the psychology of the lower animals.

